# The Sense of Agency Is More Sensitive to Manipulations of Outcome than Movement-Related Feedback Irrespective of Sensory Modality

**DOI:** 10.1371/journal.pone.0161156

**Published:** 2016-08-18

**Authors:** Nicole David, Stefan Skoruppa, Alessandro Gulberti, Johannes Schultz, Andreas K. Engel

**Affiliations:** 1 Department of Neurophysiology and Pathophysiology, University Medical Center Hamburg-Eppendorf, Hamburg, Germany; 2 Division of Medical Psychology, Department of Psychiatry, University of Bonn, Bonn, Germany; Universita degli Studi di Udine, ITALY

## Abstract

The sense of agency describes the ability to experience oneself as the agent of one's own actions. Previous studies of the sense of agency manipulated the predicted sensory feedback related either to movement execution *or* to the movement’s outcome, for example by delaying the movement of a virtual hand or the onset of a tone that resulted from a button press. Such temporal sensorimotor discrepancies reduce the sense of agency. It remains unclear whether movement-related feedback is processed differently than outcome-related feedback in terms of agency experience, especially if these types of feedback differ with respect to sensory modality. We employed a mixed-reality setup, in which participants tracked their finger movements by means of a virtual hand. They performed a single tap, which elicited a sound. The temporal contingency between the participants’ finger movements and (i) the movement of the virtual hand or (ii) the expected auditory outcome was systematically varied. In a visual control experiment, the tap elicited a visual outcome. For each feedback type and participant, changes in the sense of agency were quantified using a forced-choice paradigm and the Method of Constant Stimuli. Participants were more sensitive to delays of outcome than to delays of movement execution. This effect was very similar for visual or auditory outcome delays. Our results indicate different contributions of movement- versus outcome-related sensory feedback to the sense of agency, irrespective of the modality of the outcome. We propose that this differential sensitivity reflects the behavioral importance of assessing authorship of the outcome of an action.

## Introduction

The sense of agency is defined as the ability to experience oneself as the agent of one's own actions. It serves to distinguish actions that are self-generated from those controlled by others, and thus contributes to the subjective phenomenon of self-consciousness [1]. Even minor impairments in this ability profoundly affect an individual’s functioning in society as demonstrated by delusions of control in the healthy and diseased [2,3]. To study the sense of agency experimentally, many studies have introduced a sensorimotor mismatch between action and outcome. This disrupts the contingencies that guide us in the causal analysis of our self-efficacy within the world. This operationalization draws upon the forward model of motor control and the notion of efference copies as predictors of movement outcome [4]. If a sensory event does not match the movement, or if the predicted and actual outcome do not correspond, the event is attributed to another person or source rather than to oneself [5]. This notion has now been vastly scrutinized by numerous empirical studies.

In these studies, participants perform simple movements (e.g., finger movements), more complex movements (e.g., line drawing, moving a joystick, peg removal etc.) or button presses, while participants’ sensory feedback is manipulated [6,7]. The majority of studies have exploited feedback in the visual domain by introducing a temporal delay, spatial distortion or otherwise incongruent visual feedback [8–11]. For example, Franck and colleagues [9] displayed the image of a hand holding a joystick whose movements temporally deviated from the subjects’ own joystick movements. A few studies have manipulated auditory feedback, for example the onset or congruency of tones elicited by a button press [12–14]. Visual and auditory modulations of the sense of agency (e.g., auditory agency lagging visual agency experience or vice versa) have hardly been directly compared, and thus it remains unclear whether violations of self-agency are tolerated to similar degrees in different sensory modalities or not. More fundamental research suggests differential effects of visual versus auditory feedback on sensorimotor processing and involved neural circuits ([e.g., [15]). With respect to the sense of agency or related phenomena such as illusions of the sense of body ownership over an artificial limb (i.e., is it my body that is moving?), for example the Rubber Hand Illusion [16], there is evidence that vision can capture less salient internal signals, such as proprioception or autonomous physiological activity [17–22], inducing illusory feelings of body ownership, self-location and/ or agency—the three subcomponents of a sense of embodiment [23,24]. These feelings are evoked by congruent visuo-tactile stimulation or visuo-motor correlations. Yet, evidence on possible similar capturing effects of audition is scarce.

Importantly, the afore-mentioned studies [8–14] do not only differ in the manipulation modality but also with respect to whether they manipulated sensory feedback pertaining to the movement or the action outcome—a largely ignored matter of comparison in the field of agency investigations. A common example used to distinguish these two aspects of an action is the pressing of a light switch [22,25], where the pressing of the switch represents the movement and the change of illumination the action outcome. Metcalfe and coworkers [25] nicely coined these “proximal” and “distal” action-related consequences, respectively. Their participants played an interactive video game, in which participants moved a mouse cursor trying to catch and “pop” moving target objects. The authors either spatially manipulated the cursor’s movement on the screen (i.e., their “proximal condition”) or the degree to which the targets popped or not (i.e., “distal condition”). They found that their proximal manipulation induced a significantly bigger reduction of the participants’ sense of control than the distal condition, suggesting functional differences in the processing of feedback related to movement and outcome [25].

In this study we sought to directly compare the effects of manipulating these different aspects of action feedback on the sense of agency, taking into account effects of sensory modality, in an ecologically valid experimental setup using virtual reality technology. In different experimental conditions, we manipulated movement-related vs. outcome-related action feedback, with outcome-related feedback occurring either in the visual or auditory domain (Experiments 1 or 2). Movement-related feedback was always provided and manipulated in an embodied manner by means of our virtual reality set-up, which led a virtual hand to be processed as if it were part of the participant’s own biological body, whereas the action outcome was per definition external or disembodied. Following previously established paradigms [9,14], our participants performed cued finger movements (e.g., a single tap with the index finger akin to pressing a button). Participants received movement-related feedback by observing a virtual hand that tracked their finger movements. Outcome-related feedback about the finger tap was provided either as a click sound (Experiment 1) or a change in the color of a virtual button participants tapped on (Experiment 2). The temporal contingency (i.e. the amount of delay) between the participants’ action and (i) the tapping of the virtual hand or (ii) the auditory or visual action outcome was systematically varied. After having pressed the virtual button and experienced the click sound or color change, participants had to answer the question “Was this you or not?”. This allowed for measuring changes in participants’ attribution of agency as a function of temporal contingency between their actual finger movement and movement- or outcome-related feedback. These changes were quantified by means of the classical psychophysical Method of Constant Stimuli.

Based on the previous findings described above, we expected that the sense of agency would be lost quicker (i.e. already with smaller temporal delays) after manipulation of movement-related feedback than outcome-related feedback. We predicted effects to be independent of feedback modality, testing this prediction by implementing a visual outcome feedback in a second experiment. If anything, we expected effects to be even clearer when both movement and outcome-related feedback are presented in the same modality (e.g., like visual in Experiment 2), as this would facilitate perceptual comparison between movement and outcome [26,27].

## Material and Methods

### Participants

Fifteen right-handed adult participants with normal or corrected-to-normal vision were recruited (age 20–47 yrs., mean age 26.4 yrs., 5 male) to participate in two directly subsequent experimental runs. Before participation, each participant signed informed consent. All participants were paid an allowance of 10 €/h. The study was approved by the Ethics Committee of the Medical Council Hamburg.

### Apparatus

**Fig 1:**
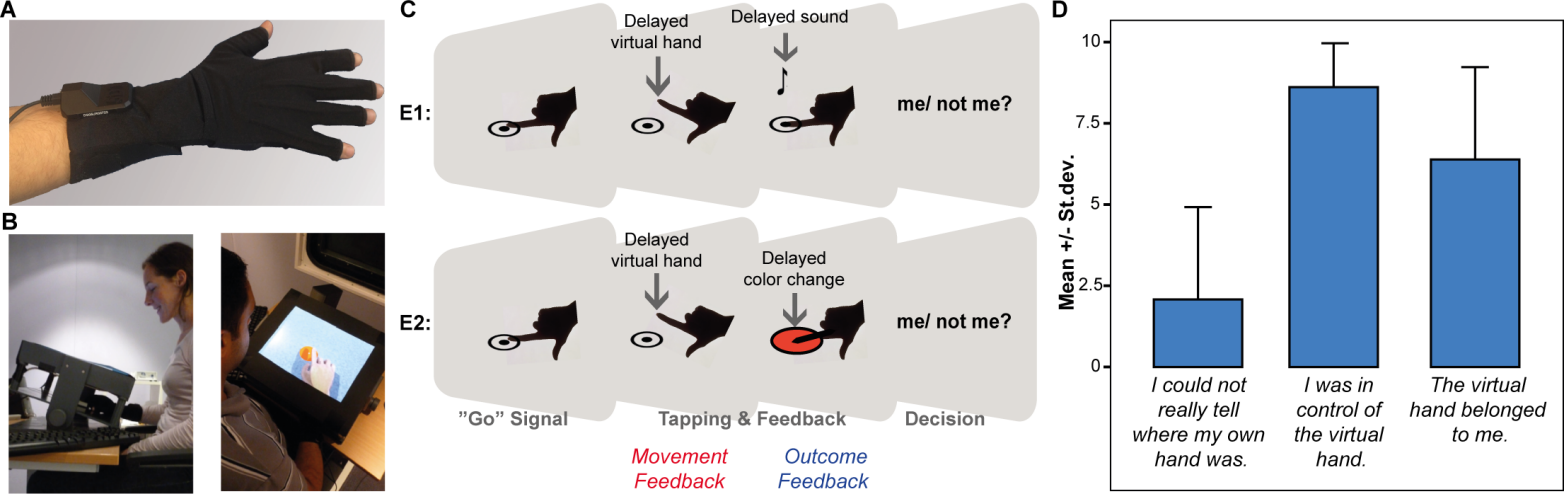
Experimental Setup and Tasks. (A) Participants wore a sensor glove to track their movements. (B) They were seated in front of an inclined computer screen and placed their gloved hand on a board underneath the screen. While performing voluntary finger movements (finger taps), participants receive real-time visual feedback from a simulated virtual hand, displayed on the screen in spatial alignment with their hidden real hand. (C) The figure schematically displays the course of a trial. Participants performed two consecutive experiments. Experiment 1 (E1): After an auditory cue, participants performed a single tap with their index finger (as if pressing a button), which evoked a click sound. Sometimes the onset of the virtual hand’s movement was delayed (feedback about movement or ‘hand’ condition). Sometimes the sound was delayed (‘outcome audio’ condition). Each trial had to be evaluated as self- or other-generated. Experiment 2 (E2): In a visual control version, participants’ taps evoked a color change in a virtual button (‘outcome color’ condition). (D) Thirteen pilot participants evaluated the set-up after a cycle of continuous tapping without delay by rating their agreement on a scale from 1 to 10 (10 = “I absolutely agree”) with respect to three questions related to ownership and agency over the virtual hand.

We employed a mixed-reality setup using an inclined computer screen (‘screen box’), which displayed a virtual hand on the same plane as the participant's real hand (Fig 1; the participants shown in this figure have given written informed PLoS consent and permission for publication). Participants placed their own hand underneath this screen (Fig 1). 3D virtual graphics were designed with Ogre3D (www.ogre3d.com) and manipulated using custom-written code. The virtual scene consisted of neutral background, a blue button and a humanoid hand. Participants wore a data glove (Data Glove 14 Ultra, 5 Dimension Technologies; Fig 1), which included movement sensors to precisely track and record finger movements and project them onto the virtual reality hand. The sensor glove coverage extended from mid-forearm to the (open) fingertips, and included 14 stretch sensors, ten of which were located on the back of the hand in two rows of five sensors, each measuring the flexion on the metacarpophalangeal or interphalangeal joints. The remaining four sensors were situated between the fingers, and measured their relative abduction. The data recorded by those sensors were used to control the movement of the virtual hand. Timing of the tapping events were additionally recorded using a touch sensor, corresponding to a small conductive plate underneath the screen, on which subjects were instructed to tap. This sensor detected a tap by closing a circuit upon touch, generating a pulse that was digitized and sent to the control software via a parallel port. Timing precision of the tap recording was only limited by the software’s read loop (~ 2 ms). The touch sensor was also used for the auditory feedback, corresponding to a click presented through speakers upon the finger's contact with the sensor. To avoid sound delays typical of standard sound cards (> 10 ms), our setup included an additional device (with an inherent delay of ~ 5 ms) to deliver a prerecorded click sound when receiving a triggering pulse from the parallel port. All components were connected to a Intel Core 2 Duo @ 3 GHz and 2GB RAM central computer running a program written in C++ on Windows XP Service Pack 3.

### Procedure

Participants were seated in front of the screen box in an acoustically shielded chamber and read standardized instructions (see below). After putting on the sensor glove, they placed their own right hand, palm downwards, under the screen so that their own hand was invisible to them. The virtual hand was then displayed and was visible several seconds before the start of each trial; it remained visible throughout the trial. Size and position, i.e., spatial alignment, of the virtual and the real hand were matched so that the system yields the illusion that the participant looked at his/her own hand through a glass sheet (Fig 1B). Each participant then underwent a brief calibration phase in order to compensate for differences in individual fit between their real hand and the sensor glove, calibrating the range of flexion and abduction. After calibration, participants performed practice trials until they felt ready for the actual task.

The complete instruction read as follows (translated by the authors): “The present study investigates how we distinguish between own actions and the actions of other people. You will sit in a sound-isolated chamber in front of a monitor. You will be asked to perform simple finger movements with your right index finger, which you will be able to track by means of a virtual hand on the screen. For this purpose, we will ask you to wear a special glove, which is equipped with movement sensors permitting us to record or track your movements. Your task will be to judge how well the movement of the virtual hand corresponds to your own movement. Please keep your right hand as still as possible. Three beep tones will signal the beginning of each trial. After the third beep, briefly lift your index finger and lower it back again (the experimenter will show you how). At the end of your movement, you will hear a feedback tone (on the second run, the color of a disc on the screen will change instead). Another person will carry out the same task on a second computer outside the cabin. The movements of the virtual hand can sometimes be your own or belong to the other person. It is your task to decide! Thus, please carefully observe the hand’s movements on the screen, especially with respect to the onset and offset of your own movement! Thus, also observe whether the end of your finger movement corresponds to the onset of the feedback tone (or the colour change)! After each trial, you will be asked to judge by means of the corresponding button on the keyboard: Was this me or another person? y =, "yes", n = "no". Please, react fast! You only have 3 sec to decide and start your movement.”

In a related, continuous tapping study, which used the same set-up and was conducted immediately before the present experiments, thirteen pilot participants rated their agreement on three items taken from a questionnaire originally devised for the Rubber Hand Illusion [23]. These pilot data revealed that participants on average experienced ownership and agency over the virtual hand while not disembodying their own hand (Fig 1).

### Tasks

Each participant completed two consecutive experiments (Fig 1). In the first experiment, participants performed cued movements with their right index finger, while observing the finger movement of the virtual hand. Three beeps signaled the beginning of a trial. At the end of the third beep, participants were instructed to lift their index finger and perform a single tap. The participants’ movement was represented by a virtual hand, which tapped onto a virtual button (Fig 1). Contact with the touch sensor during the tap evoked a click as auditory action outcome. A temporal delay was introduced between the participants’ own movement and (i) the movement of the virtual hand (hereafter referred to as ‘hand’ condition) *or* (ii) the click tone to manipulate the sense of agency (hereafter referred to as ‘outcome audio’ condition). There was always only one type of feedback manipulation per trial. There were six delay levels (0, 100, 200, 300, 400, 600 ms, occurring in 30 trials each) per feedback condition, which were determined as a result of piloting and based on previous evidence [8,9,11,14,28]. The order of delays and feedback types was pseudo-randomized within and across participants. Participants were instructed to always pay close attention to both (i) the onset of their own and the virtual hand's movement (i.e., matching or mismatching), and (ii) whether the onset of the auditory feedback matched the offset of their own finger movement. Participants rated each trial by pushing one of two buttons on an external keyboard with their left hand (Was it me? ‘Yes’/ ‘No’). They were instructed to react quickly during the entire experiment. The time allowed for initiating a movement and for reporting the agency percept was limited to three seconds, after which the trial was aborted.

To test that differences in the sense of agency for the different types of feedback were not merely due to differences in processing delays in the visual versus auditory domain, participants performed a control experiment in a second block. Here the ‘hand’ condition was the same as above. That is, there was a matching/ mismatching onset of the virtual hand's and the participant's own movement. Yet, instead of hearing a click as a result of each finger movement, the virtual button, on which participants tapped, changed its color from blue to red upon collision of the participant's real finger with the touch sensor (hereafter referred to as ‘outcome color’; Fig 1C). This color change was manipulated using the same aforementioned six delays. Participants were explicitly instructed to pay close attention to the matching of the onset of their own and the virtual hand's movement, and the onset of the color change and the offset of their own finger movement.

In both experiments, in trials with long or evident delays of the virtual movement onset (e.g., when the virtual hand moved 600 ms after the participant’s actual movement onset), a few very quick participants sometimes reached the end of their movement before the virtual hand had even started to move. Such trials were eliminated from further analysis.

### Data Analysis

The proportion of ‘me’ decisions across trials of each condition (‘hand’, both ‘outcome’ conditions) was our dependent variable, and was calculated per delay level for each participant, in both experiments. The proportion ‘me’ data were subjected to a 3-way, repeated-measures ANOVA with the factor delay duration (100, 200, 300, 400 and 600 ms), feedback type (‘outcome’ and ‘hand’), and experiment (Experiments 1 and 2).

## Results

**Fig 2:**
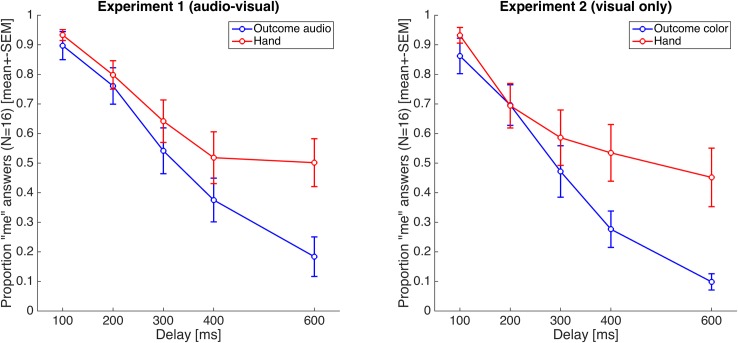
Sense of agency as a function of delay across conditions (i.e., feedback types) and experiments. Perceptual reports of self-agency (i.e., proportion ‘me’) are plotted as a function of delay (i.e., 0, 100, 200, 300, 400, 600 ms) of the visual feedback about the movement itself (‘hand’ condition) or the feedback about the movement outcome (Experiment 1: ‘outcome audio’, Experiment 2: ‘outcome color’ condition). Data are mean values over all participant, error bars represent one standard error of the mean.

Across experiments, participants’ sense of agency decreased with longer delays irrespective of feedback type (Fig 2, main effect of delay duration: F(1,300) = 43.48, p<0.001). Importantly, participants’ probability of self-agency reports (“it was me”) decreased more quickly as a function of increasing delay when the delays occurred in the anticipated sensory outcome (‘outcome’ conditions) than when delays occurred in the virtual movement onset (‘hand’ conditions) (Fig 2; main effect of ‘outcome’ vs. ‘hand’ delay: F(1,300) = 19.39, p<0.001; interaction between delay duration and feedback type: F(3,300) = 3.12, p<0.02). In other words, delay duration had a more pronounced effect on self-agency reports in the ‘outcome’ than the ‘hand’ condition. This was not significantly different across experiments (main effect of experiment: F(1,300) = 2.85, p>0.09; interaction between experiment and delay duration: F(4,300) = 0.13, p>0.9; interaction between experiment and feedback type: F(4,300) = 0.24, p>0.6; 3-way interaction: F(4,300) = 0.015, p>0.9). Post-hoc comparisons between conditions did also not reveal significant differences between ‘outcome’ or ‘hand’ conditions across experiments (Tukey-Kramer, N.S.).

In sum, our results therefore show that participants’ sense of agency was more sensitive to feedback delays when these delays affected the outcome of their action than when they affected visual feedback about the effector of the action (i.e. the participants’ hand).

## Discussion

We sought to disentangle modulations of the sense of agency for body- or movement-related feedback (displayed as a virtual hand following the participants’ hand with a delay) vs. external outcome (a delayed sound occurring as a consequence of the movement). Contrary to our predictions based on previous findings, participants were more sensitive to delays of outcome as opposed to movement. This was not due to a fundamental processing advantage in the auditory vs. visual domain, as the same pattern of results was obtained in a visual-only version of the experiment, in which the sound manipulation was replaced by a visual change in the environment. Our results suggest conceptual and phenomenological differences between the perception of agency based on movement or action outcome.

### Agency for Outcome- vs. Movement-Related Sensory Feedback

The important distinction between agency for movement vs. external outcome itself has only rarely been addressed empirically (e.g., [25]). In contrast to our own results, the authors found that their movement-related—or, what they called “proximal”—manipulation induced a significantly bigger reduction of the participants’ sense of control. There are a number of substantial differences between the former and the present study. First, the former study employed more coarse-grained spatial distortions (as proximal condition) *vs*. oddball distortions (i.e., an expected event did not happen; as distal condition), whereas we employed a series of fine-grained temporal distortions of feedback onset to manipulate proximal *and* distal feedback in different conditions. Second, our proximal or movement-related feedback was provided by means of a virtual hand displaying the movements of the participants, while [25] investigated extracorporeal effects in both proximal and distal conditions. As such, Metcalfe and colleagues’ proximal manipulation may not be as different from our distal manipulation at the level of initial, direct action effects. We, thus, fully support the authors’ proposal that different psychological profiles—and possibly functional mechanisms—are associated with movement- and outcome-related cues to agency.

Our current results might also be compatible with the idea that for agency judgments based on embodied, internal or movement-related cues, the results of an efference-copy-based sensorimotor matching process might not always be consciously available [5]. As Synofzik and colleagues [5] put it, “When subjects plan, monitor and perceive their own actions and the corresponding effects, they often do not primarily represent them in motor-related terms (e.g. their spatio-temporal pattern), but in intentional and perceptual terms (e.g. their underlying goals)”. Such representations may lack the temporal precision required to detect slight delays of the virtual hand (see below for further discussion on the effects of representing the virtual hand).

In addition, dramatically different psychological (or ‘phenomenological’) profiles are also to be expected if operationalizations of agency employ body shapes as opposed to non-corporeal operationalizations such as cursor movements [29]. As such, the specifics about how proximal and distal manipulations are implemented, for example, with respect to body form or whether they happen in an embodied vs. disembodied context, need to be considered.

### Effects of the Virtual Hand on Agency Judgments

There is an ever-growing body of work demonstrating the psychological and physiological embodiment of virtual or rubber hands or bodies, even tools [23, 24, 30–32]. Unfortunately, there also is an ongoing debate about what the term “embodiment” actually refers to. Our aim in the current study was not to participate in this debate. Here, embodiment is primarily understood in relation to our virtual reality application or 3D-simulated immersive environment, in which the virtual hand acted as a placeholder for the participant’s real hand, observed from a first person perspective. The participant had the ability to act from that perspective so that the virtual hand was processed as if it were part of the participant’s own biological body, allowing him to recognize himself as the author of the virtual movements [24].

As such, one possibility to explain our pattern of results is that embodiment of the virtual hand may have rendered the detection of the movement-related feedback delay more difficult (see the relatively low sensitivity for 600-ms delays in the “hand” condition). This very reliable effect was almost identically present in both our experiments. Did embodiment of the virtual hand somehow perturb or interfere with the participants’ own movement representation? Neuropsychological evidence suggests that misrepresentation of a body part might hinder the motor system from programming appropriate commands and from predicting sensory consequences of movements pertaining to this part. For example, participants with anomalous anatomical features show differences from typical participants in handedness tasks as well as visual processing of both biological motion and body shape [33]. Moreover, exciting recent evidence about the link between embodiment and movement representation comes from studies of body ownership illusions such as the Rubber Hand Illusion [16]. This illusion, which is usually evoked through simultaneous visuo-tactile stimulation of a participant’s own hand and the rubber hand, can also be evoked when the artificial body part is seen to move synchronously with the real hidden counterpart [34–36]. In fact, even larger body parts can be perceived as part of the own body when they are seen to move in a way compatible with the self’s movement intentions [37]. In turn, experiencing body ownership over an alien body can lead to experiencing the sense of agency over the alien body’s actions [38]. In contrast, the RHI is cancelled out when the rubber hand is replaced by a non-body object, and discontinuous bodies are less likely to evoke the senses of agency and body ownership [20,39], suggesting that “some form of ‘body model’ serves a perceptual filter” for embodiment and its subcomponents [40]. Interestingly, experiments using variants of the RHI in which participants act with the rubber hand have revealed a double-dissociation between body ownership and agency, but also an increase in the sense of agency during body ownership [23,36,41–42]. Our results are broadly consistent with the novel and interesting conceptual distinction between “external agency” (i.e., over mouse control or cursors) and “body agency” (i.e., over limbs or moving fake hand) (as, e.g., suggested by [36]). Experiments studying the link between the senses of embodiment and agency will allow to further investigate this distinction.

### The Role of Sensory Modality for Movement- vs. Outcome-Related Feedback

The sense of agency can be considered a special case of perceived causality between a movement/ action and a temporally contingent sensory effect (e.g., light as effect of pushing a switch) [43]. As such, the sense of agency also represents a special case of cross-modal interaction [26]: It involves the integration of internal signals associated with movement execution and exteroceptive feedback signals. Typically, researchers exploited visual or auditory feedback signals. Differential modality-specific modulations of the sense of agency remain a matter of conjecture. Our results indicate higher sensitivities for outcome- vs. movement-related feedback, and this pattern of results appeared whether the same (visual-only experiment) or different modalities were tested (visual-auditory experiment), suggesting a general modality-independent processing advantage in terms of awareness for action outcome compared to movement dynamics.

Sensory information other than auditory or visual, such as proprioception, has also been crucially implicated in the sense of agency [44,45], especially in relation to movement-related operationalizations. Internal signals such as proprioception are more difficult to manipulate in a laboratory setting; therefore, experiments typically tackled the sense of agency by manipulation of reafferent sensory information [7], unless they examined rare cases of deafferented patients [44,45]. Balslev and colleagues [44], for example, showed that when proprioceptive signals were unavailable, a chronically deafferented patient was impaired in discriminating self- from computer-produced visual feedback based on the timing of movement. It has now been repeatedly demonstrated that sensitivity to proprioceptive or other internal signals is limited and often overruled by exteroceptive senses, especially vision. This might have also accounted for our main result (sensitivity to outcome delay > movement-related delay). Participants might have been less sensitive to the movement delay because the agency judgments in the ‘hand’ condition were based on comparisons between proprioceptive information, efferent motor signals and visual input, while outcome delays were predicted by exteroceptive cues in the visual and tactile senses (i.e., collision of own finger with touch sensor). This could have rendered the detection of the movement-related feedback delay in the virtual hand more difficult than delay in the outcome-related feedback.

### Alternative Accounts, Limitations and Conclusions

Unfortunately, our conclusions are limited by the fact that we did not implement an auditory-auditory control experiment, in which the movement-related feedback would have been provided (and manipulated) by means of some sort of auditory feedback in the absence of vision. Although possibly difficult to achieve, this certainly represents an innovative and interesting future research endeavor, enabled for example through recent technological developments such as sonification systems [46].

Second, in contrast to our present finding, it has been shown that participants detected a false visual feedback already at quite brief delays (e.g., </ = 150 ms) [8,9,28,47,48]. In contrast to these studies that implemented only one kind of feedback (e.g. delayed virtual movement only *or* delayed tone), here participants were instructed to continuously monitor both movement and outcome and compare those with respect to their own movement onset (in case of the ‘hand’ condition) and offset (‘outcome’ condition). The design of this task did therefore not allow identifying upon which cue the agency judgment was actually based.

Third, our experimental conditions (‘hand’ or ‘outcome’) also differed with respect to temporal characteristics including duration and order. For example, the virtual hand’s motion always occurred before the sound/ color change, as a result of the design of the experiment. Moreover, despite being instructed to pay attention to the critical onset in all feedback types, the longer duration of the movement and the resulting sensorimotor dynamics might have masked detection of delays in movement onset. We cannot rule out that such differences in stimulus timing contributed to our main result. Nonetheless, this limitation represents an ecologically valid approach reflecting the real world: action consequences inherently occur after movements and are naturally in the focus of attention in our everyday, effortless goal-oriented behavior.

Our experimental approach may help to characterize the level at which the sense of agency arguably plays its most important role: ascertaining authorship of the consequences of our actions. At this level, disturbances could result from unplanned influences of the environment or other people interfering with our action, while our hand moves most often the way we intended it to. Thus, mismatches between an intention and the outcome of an action should be detected as precisely as possible: after all, acting out our intentions is the reason why we do any action.
